# Resting-State EEG Analysis Characterizes the Signature of *CACNA1A*-and GAA-*FGF14*-Related Channelopathies

**DOI:** 10.1007/s12311-025-01924-w

**Published:** 2025-11-15

**Authors:** Raphael Angerbauer, Iris Unterberger, Wolfgang Nachbauer, Matthias Amprosi, Sylvia Boesch, Matteo Cesari, Elisabetta Indelicato

**Affiliations:** 1 Department of Neurology, Medical University Innsbruck, Innsbruck, Austria; 2 Center for Rare Movement Disorders Innsbruck, Department of Neurology, Medical University Innsbruck, Anichstrasse 35, Innsbruck, 6020 Austria

**Keywords:** Resting-state EEG, Connectivity analysis, CACNA1A, FGF14, Spinocerebellar ataxia type 27B

## Abstract

Cerebellar ataxia frequently results from ion channel dysfunction, with *CACNA1A-* and GAA-*FGF14*-related diseases representing two of the most prevalent genetic etiologies. While both disorders may share overlapping clinical features, their pathophysiology remain distinct and incompletely understood. Advanced resting-state electroencephalogram (rsEEG) analysis is an established methodology to assess cortical dynamics and network dysfunction in brain disorders. We applied advanced rsEEG analysis to identify disease-specific electrophysiological patterns in *CACNA1A-* and GAA-*FGF14*-related diseases. Routine scalp EEG examinations from genetically confirmed patients were retrospectively collected at the Department of Neurology of the Medical University Innsbruck. EEGs from matched healthy controls were retrieved from a publicly available database. Using a Bayesian hierarchical modeling framework, we analyzed spectral bandpower and functional connectivity metrics. Compared to healthy controls, *CACNA1A* patients (*n* = 29) exhibited significantly increased theta-band power and reduced alpha peak frequency across all brain regions. Additionally, they showed enhanced functional connectivity in both the delta/theta and gamma frequency bands. In contrast, findings in the GAA-*FGF14*–related group (*n* = 15) largely overlapped with those of healthy controls, with only mild alterations characterized by increased beta power in posterior regions and a hyperconnectivity pattern in the alpha band. *CACNA1A*-related disease is associated with widespread cortical network dysfunction, aligning with the clinical observation of frequent cognitive and neuropsychiatric symptoms—unlike the pure motor presentation seen in GAA-*FGF14*–related disease. Advanced rsEEG analysis allows for the non-invasive and repeatable detection and quantification of these alterations, holding promise for the development of surrogate markers for rare channelopathies.

**Supplementary Information:**

The online version contains supplementary material available at 10.1007/s12311-025-01924-w.

## Introduction

Ion channel dysfunction is a recurring pathophysiological mechanism in inherited cerebellar disorders [1]. Cerebellar disorders associated with channelopathies typically present with chronic and episodic neurological symptoms [2]. *CACNA1A* [MIM 601011] and GAA-*FGF14* [MIM 620174] related disorders recapitulate these features and are among the most frequent molecular etiologies of inherited ataxias [2, 3].

*CACNA1A* is a bicistronic gene which encodes both the pore-forming α1A-subunit of the neuronal calcium channel Cav2.1 and the transcription factor α1ACT which drives the early development of the Purkinje cells [4]. *CACNA1A*-related disorders are rare, and their estimated incidence rate is 1:11,700 [5]. However, *CACNA1A* variants emerged as the most common monogenic etiology in a large global cohort of patients with cerebellar ataxias due to non-expansion variants [2]. Typical symptoms encompass developmental delay in early-onset cases, a usually mild chronic cerebellar syndrome, as well as behavioral issues and neuropsychiatric disturbances [6]. Episodic manifestations include migraine with hemiplegic aura, episodic ataxia, and epilepsy [6].

*FGF14* encodes a member of the fibroblast growth factor family. *FGF14* directly binds and regulates the function of both potassium and voltage-gated sodium channels, thus influencing membrane potential and synaptic transmission [7]. Recently, a pathologically expanded deep intronic GAA-repeat in the *FGF14* gene has been associated with an adult-onset ataxia with frequent episodic symptoms, known as spinocerebellar ataxia type 27B (SCA27B) [8]. This entity overlaps the phenotype of the *CACNA1A* disorder known as “episodic ataxia type 2”.

Channelopathies may affect synaptic potentials, the source of electroencephalogram (EEG) signals. In non-expansion *CACNA1A* disorders, seizures and epileptiform activity in EEG are typically observed in childhood, before onset of hemiplegic migraine/episodic ataxia and abate later in life [9, 10]. Nonetheless, adults may show recurring non-epileptic EEG changes [9]. In a previous work of our group based on visual analysis of routine EEGs, we observed especially a lateralized, intermittent slowing in the temporal region [9]. This unspecific finding occurred at a significant higher frequency in genetically confirmed *CACNA1A* patients as compared with the control group, consisting of phenocopies with episodic ataxia or hemiplegic migraine but negative genetic testing for *CACNA1A*. Currently, a number of these controls have been tested positive for GAA-*FGF14* disease (seven out of 18 “*CACNA1A* phenocopies” from [9], 39%).

Beyond the routine naked-eye examination for detecting epileptic activity or regional changes, advanced analytical approaches can leverage a much wider information from resting-state EEG (rsEEG) signals [11]. Indeed, rsEEG reflects the default mode network, which underlies many non-task-related functions including memory formation [12]. A common approach to analyzing rsEEG involves quantifying the relative contribution of oscillatory activity within specific frequency bands to the overall signal. Alterations in the power of these bands can reflect or even predate clinical milestones in neurodegenerative disorders. For instance, an increased power in lower frequency bands is a predictive marker of cognitive dysfunction in Parkinson´s disease [13, 14]. In addition to spectral power analysis, rsEEG can also be used to assess functional connectivity between brain regions by evaluating signal similarity across spatially distant electrodes. Distinct patterns of altered functional connectivity are associated with specific underlying pathophysiological mechanisms across different neurodegenerative disorders [15, 16].

In the present study, we investigated whether advanced, objective rsEEG analysis can identify disease-specific electrophysiological signatures in *CACNA1A*- and GAA-*FGF14*-related disorders—two channelopathies with overlapping clinical phenotype.

## Patients and Methods

### Patient Selection

Adult patients with cerebellar ataxia due to genetically confirmed non-expansion *CACNA1A*-disease or GAA-*FGF14*-related ataxia were recruited from the Center for Rare Movement Disorders of the Medical University of Innsbruck. Control subjects were retrieved from a publicly available dataset [17].

## EEG Analysis

### Acquisition

Patients EEGs were recorded using a Natus Schwarzer epas 29 amplifier system in the EEG laboratory of the Department of Neurology, Medical University of Innsbruck. EEGs were recorded during wakefulness, between 9 a.m. and 3 p.m, using a standard 10–20 electrode placement system with 24 electrodes at a sampling rate of 256 Hz. Electrode impedances were kept below 10 kΩ. Patients were repeatedly instructed to remain still with eyes closed for 150 s. Interspersed periods with eyes open of the same length were then removed from the analysis. We excluded EEGs performed during or up to one week after spell episodes. Controls EEGs from [17] were recorded via a BioSemi ActiveTwo 64-channel system (following the 10–20 electrode placement system) at a sampling rate of 1024 Hz.

### Preprocessing

To ensure comparability across recordings, all EEGs underwent the same preprocessing conducted using Python (Version 3.11) and the MNE library [18]. Signals were bandpass filtered between 0.5 and 40 Hz using a Butterworth filter and notch-filtered at 50 Hz to remove power line interference. The continuous EEG signal was segmented into 2-second epochs, a duration optimized for spectral connectivity analysis [19]. Epochs contaminated with artifacts were identified and rejected using an independent component analysis (ICA)-based artifact removal algorithm [20]. ICA components correlating with the electrooculogram were excluded using adaptive z-scoring to mitigate eye movement artifacts. The cleaned signals were the re-referenced to the average of all channels to remove common noise sources.

## Bandpower

Power spectral density (PSD) was estimated for each epoch using the Welch method with a hamming window size of 1 s without overlap. Absolute power was calculated for the following frequency bands: delta (0.5–3 Hz), theta (3–7 Hz), alpha (7–12 Hz), beta (12–30 Hz), and gamma (30–40 Hz), by integrating the PSD within each respective band. Relative power was obtained by normalizing absolute bandpower to the total power across all frequency bands. For statistical analysis, relative power values were averaged over epochs and grouped into anatomical regions: frontal (Fp1, Fp2, Fz, F3, F4, F7, F8), central (C3, C4, Cz), parietal (P3, P4, Pz), and occipital (O1, O2).

### Connectivity

Functional connectivity was assessed using the weighted Phase-lag Index (wPLI), a metric robust against volume conduction effects [21]. wPLI was computed for all electrode pairs per epoch and averaged across epochs to obtain an overall connectivity measure. To account for spurious connections and extract meaningful network properties, a minimum spanning tree (MST) was constructed from the wPLI-weighted graph. The MST retains the strongest functional connections while preventing loops [22]. The following MST metrics were analyzed: Diameter (Longest path in the MST, normalized by the number of edges), Leaf fraction (Proportion of nodes with a single connection) and Maximum betweenness centrality (MBC; Fraction of shortest paths passing through the most central node). Longer Diameter indicates a less efficient network, while a higher leaf fraction value hints at a more tree-like structure. In a graph with high MBC connections between nodes usually need to pass through a central “hub” node, making connections shorter but decreasing the robustness of the network.

## Statistics

### Bayesian Hierarchical Model

In conventional statistical approaches, testing a large number of variables would require correction for multiple comparisons, increasing the risk of overlooking meaningful effects. Moreover, performing separate tests for each variable ignores the natural relationships between them. For example, relative bandpower values across different brain regions are spatially correlated, and connectivity measures such as MST diameter, leaf fraction, and maximum betweenness centrality are mathematically interdependent. To appropriately account for these relationships, we employed Bayesian hierarchical models. These models allow for the joint modeling of multiple related variables while sharing information across parameters, reducing the risk of false positives and eliminating the need for correction for multiple comparisons [23].

All models were implemented in Python using the PyMC3 library [24]. Continuous variables were standardized to improve prior selection and model fitting. Weakly informative priors were used to regularize estimates while allowing the data to drive inference. Priors were initially set to a standard normal distribution and refined through prior predictive checks. Model validity was assessed by comparing observed data distributions to posterior predictive samples to ensure adequate fit. A coefficient was considered statistically significant if the 95% highest-density interval (95% HDI) of the posterior distribution did not include zero. This model is further described in an accompanying methods paper [25].

### Model for Relative Bandpowers

Relative bandpower values are inherently dependent because they must sum to one within each subject. To address this constraint, we transformed the five relative power values into the logarithm-transformed ratios of two or more bandpowers [26], and re-transformed the results back into relative powers afterwards. Specifically, the log-transformed slow-to-fast ratio [(delta + theta)/(alpha + beta)], was chosen due to its well-established relevance to EEG spectral features [27, 28].

For each brain region, the vector of log-ratio values was assumed to follow a multivariate normal distribution, with the mean of each log-ratio estimated using a linear model. Predictor variables included group, age, and sex. To facilitate interpretation, the posterior distributions of these coefficients, which are specific to the log-ratios, were back-transformed into relative power values, allowing us to assess their significance in predicting relative power.

### Model for Connectivity Measures

Connectivity measures were modeled jointly to account for their inherent dependencies. Since overall wPLI values are bounded between 0 and 1, they were logit-transformed to allow for appropriate statistical modeling. The three MST-derived metrics (diameter, leaf fraction, and maximum betweenness centrality) are highly correlated, as they are different representations of the same underlying network structure. To explicitly incorporate this correlation structure, we simulated 10,000 fully connected weighted graphs, calculated the minimum spanning tree (MST) for each, and then computed the associated connectivity measures.

For each frequency band, the vector containing overall wPLI and the three MST-derived metrics was assumed to follow a multivariate normal distribution, with its covariance structure informed by the previously determined correlation matrix. The mean of each variable was estimated using a linear model, with group, age, and sex as predictor variables. To regularize the estimates and prevent extreme values, coefficients for each frequency band were modeled to be distributed around a common coefficient.

## Results

We collected single EEG recordings from 29 adult subjects with cerebellar ataxia due to non-expansion *CACNA1A* disease and from 15 subjects with GAA-*FGF14*-related ataxia. Table 1 summarizes the demographic information of the participants. Patients with GAA-*FGF14*-disease were significantly older on average than those with *CACNA1A* variants (67.9±8.4 versus 40.2±16.84 years, *p* < 0.001 at Student’s t-test). The severity of the chronic cerebellar syndrome was comparable in the two groups and rather mild (see Table 1). Formal neuropsychological testing revealed cognitive deficits in the majority of *CACNA1A* patients (19 out of 22 versus 5 out of 14 in the GAA-*FGF14* group; *p* < 0.01 at Fisher’s exact test). Ten *CACNA1A* patients suffered from psychiatric comorbidities (anxiety, depression, adaptation disorder, attention deficit hyperactivity disorder, personality disorder, schizophrenia). Three GAA-*FGF14* patients were diagnosed with anxiety disorder. None of the patients from this adult cohort had epilepsy. At the time of EEG recording, 14 out of 29 (48%) *CACNA1A* patients and 4 out of 15 (27%) GAA-*FGF14* patients were receiving centrally acting medications (*p* = 0.21, Fisher’s exact test). The most common medication at the time of EEG recording was acetazolamide, used by 9 out of 29 *CACNA1A* patients and 2 out of 15 GAA-*FGF14* patients (*p* = 0.36, Fisher’s exact test). Single EEG recordings from 111 healthy subjects were retrieved from a publicly available dataset [17]. From this collective, we randomly selected a subset of 30 subjects individuals weighted by their similarity to patients with *CACNA1A* variants. The remaining subset (*n* = 81) was used to determine the priors for the model coefficients.

**Table 1:** Demographic characteristics and medication use

	CACNA1A	GAA-FGF14	HC	*P* value
Females (n., %)	9 (31.0%)	4 (26.7%)	9 (30.0%)	0.96
Age (years)	40.2±16.8	67.9±8.4	42.5±16.6	**< 0.001**
SARA score*	7 (IQR 4–10)	9 (IQR 7–11)	--	0.08
Psychiatric comorbidities (n., %)	10 (34.5%)	3 (20%)	--	0.48
Cognitive deficits**	19/22	5/14	--	**< 0.01**
*Medication use (n.*,* %)*				
4-Aminopyridine	0 (0.0%)	1 (6.7%)	--	0.73
Acetazolamide	9 (31.0%)	2 (13.3%)	--	0.36
Antipsychotics	2 (6.9%)	0 (0.0%)	--	0.78
Benzodiazepines	2 (6.9%)	0 (0.0%)	--	0.78
Flunarizine	2 (6.9%)	0 (0.0%)	--	0.78
Valproic acid	1 (3.4%)	0 (0.0%)	--	1.0

Statistically significant comparisons are highlighted in bold. *HC* healthy controls, *IQR* interquartile range, *SARA* scale for the assessment and rating of ataxia. * SARA scores at the time of the EEG were available for 20/29 CACNA1A- and for 10/15 GAA-FGF14 patients; statistical comparison of SARA scores was performed by means of mann-whitney-U test. ** As detected by formal neuropsychological testing which was performed at the time of the EEG in 22 CACNA1A- and 14 GAA-FGF14 patients

### Bandpower Analysis

**Fig. 1:**
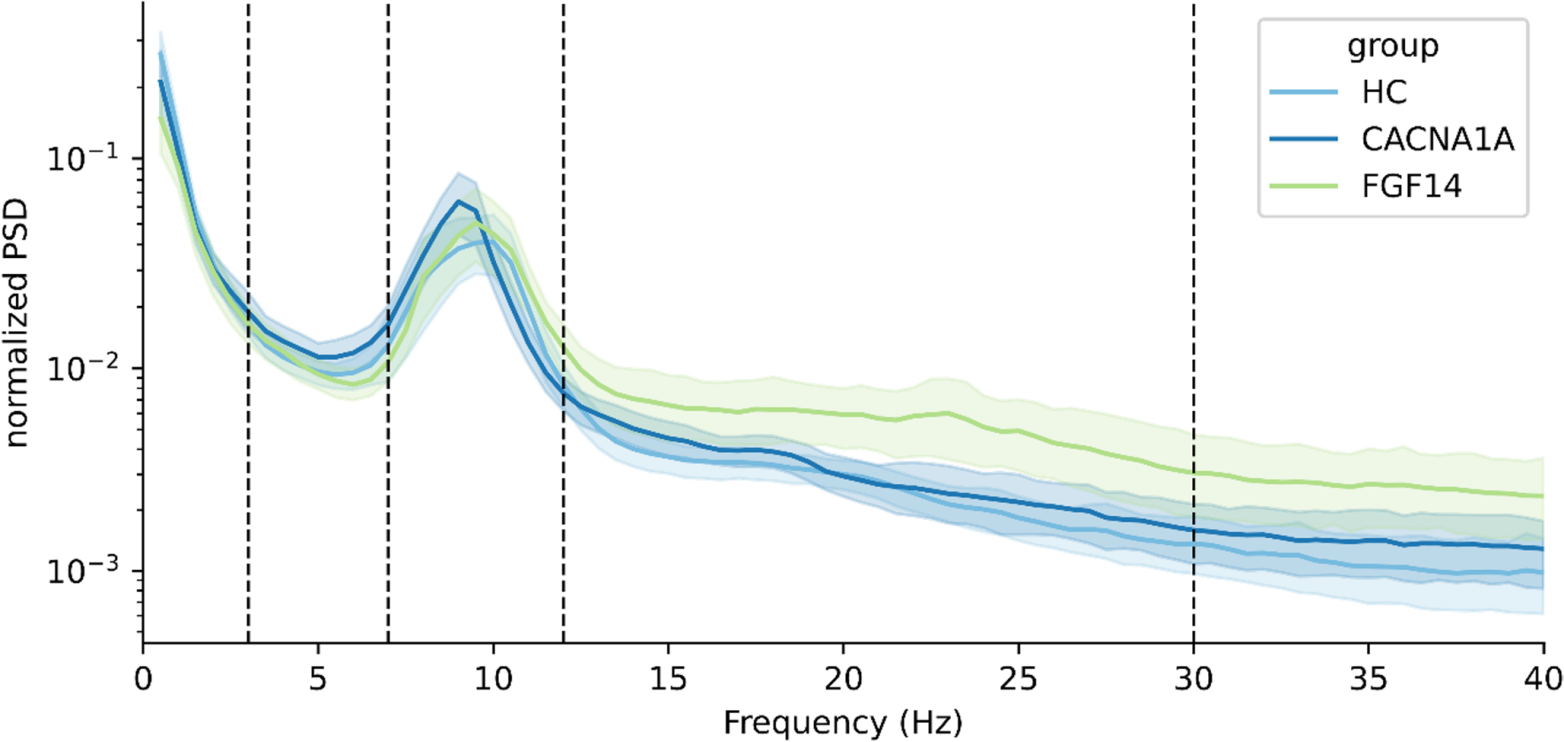
Normalized overall power-spectral densities. The spectrogram is subdivided into 5 regions corresponding to the EEG bands delta, theta, alpha, beta and gamma with the borders indicated by dashed lines. Normalized PSD values were log-transformed. HC: healthy controls

**Fig. 2:**
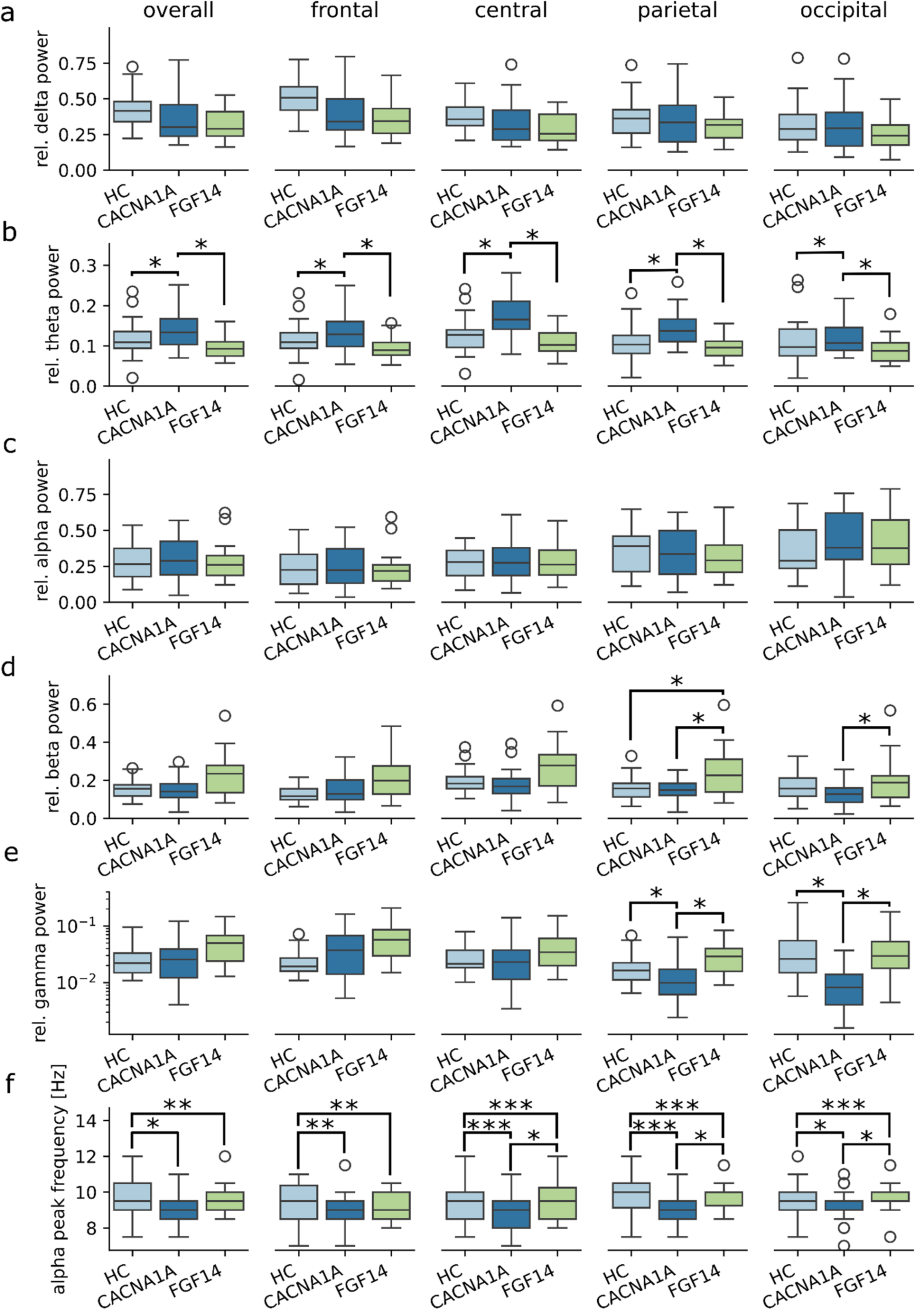
Results of relative bandpower analysis. Relative bandpowers of the delta (**a**), theta (**b**), alpha (**c**), beta (**d**) and gamma (**e**) bands and the alpha peak frequency (**f**) measured in frontal, central, parietal and occipital channels or averaged over the whole scalp (overall). Relative gamma power is displayed as log-transformed only for visualization purposes. HC: healthy controls, *: *p* < 0.05, **: *p* < 0. 01, ***: *p* < 0.001

The estimated normalized power spectra averaged over all channels and within each group are shown in Fig. 1 and the results of the bandpower analysis are reported in Table 2. Considering power spectra averaged over all channels, *CACNA1A* patients displayed (i) an increased power in the theta band, as well as (ii) a reduced alpha peak frequency compared to both the controls and the GAA-*FGF14* patients. This latter finding was already evident at visual inspection of the spectrogram (Fig. 1) and was statistically significant in the further analysis (Fig. 2f).

**Table 2:** Bandpower analysis findings

		CACNA1A vs. HC	FGF14 vs. HC	CACNA1A vs. FGF14
band	location	MD [HDI]	MD [HDI]	MD [HDI]
***delta***	***overall***	−0.03 [−0.08, 0.02] (ns)	−0.04 [−0.09, 0.02] (ns)	0.01 [−0.06, 0.08] (ns)
	***frontal***	−0.04 [−0.09, 0.01] (ns)	−0.05 [−0.11, 0.01] (ns)	0.01 [−0.06, 0.08] (ns)
	***central***	−0.04 [−0.09, 0.01] (ns)	−0.05 [−0.1, 0.01] (ns)	0.01 [−0.06, 0.08] (ns)
	***parietal***	−0.03 [−0.07, 0.03] (ns)	−0.04 [−0.09, 0.02] (ns)	0.01 [−0.06, 0.08] (ns)
	***occipital***	−0.03 [−0.08, 0.02] (ns)	−0.04 [−0.1, 0.01] (ns)	0.02 [−0.05, 0.08] (ns)
***theta***	***overall***	**0.03 [0.01**, 0.06] (*)	−0.01 [−0.03, 0.01] (ns)	**0.04 [0.01**, 0.07] (*)
	***frontal***	**0.03 [0.01**, 0.05] (*)	−0.0 [−0.02, 0.01] (ns)	**0.04 [0.02**, 0.06] (*)
	***central***	**0.04 [0.02**, 0.06] (*)	−0.01 [−0.03, 0.01] (ns)	**0.05 [0.03**, 0.08] (*)
	***parietal***	**0.04 [0.02**, 0.05] (*)	−0.01 [−0.02, 0.01] (ns)	**0.04 [0.02**, 0.06] (*)
	***occipital***	**0.02 [0.01**, 0.04] (*)	−0.01 [−0.03, 0.01] (ns)	**0.03 [0.01**, 0.06] (*)
***alpha***	***overall***	0.01 [−0.06, 0.08] (ns)	0.02 [−0.07, 0.1] (ns)	−0.01 [−0.11, 0.1] (ns)
	***frontal***	−0.0 [−0.05, 0.05] (ns)	0.01 [−0.06, 0.08] (ns)	−0.01 [−0.09, 0.07] (ns)
	***central***	−0.0 [−0.06, 0.06] (ns)	0.03 [−0.05, 0.11] (ns)	−0.03 [−0.12, 0.06] (ns)
	***parietal***	0.0 [−0.06, 0.06] (ns)	0.0 [−0.08, 0.08] (ns)	−0.0 [−0.09, 0.09] (ns)
	***occipital***	0.04 [−0.02, 0.11] (ns)	0.03 [−0.04, 0.11] (ns)	0.01 [−0.08, 0.1] (ns)
***beta***	***overall***	−0.01 [−0.04, 0.03] (ns)	0.03 [−0.02, 0.07] (ns)	−0.03 [−0.09, 0.02] (ns)
	***frontal***	−0.0 [−0.02, 0.02] (ns)	0.03 [−0.0, 0.06] (ns)	−0.03 [−0.07, 0.0] (ns)
	***central***	−0.0 [−0.03, 0.02] (ns)	0.02 [−0.02, 0.06] (ns)	−0.03 [−0.07, 0.02] (ns)
	***parietal***	−0.0 [−0.03, 0.02] (ns)	**0.03 [0.0**, 0.07] (*)	**−0.04 [−0.08**, −0.0] (*)
	***occipital***	−0.02 [−0.04, 0.0] (ns)	0.02 [−0.01, 0.05] (ns)	**−0.04 [−0.08**, −0.01] (*)
***gamma***	***overall***	−0.0 [−0.01, 0.01] (ns)	0.0 [−0.01, 0.02] (ns)	−0.01 [−0.02, 0.01] (ns)
	***frontal***	0.01 [−0.0, 0.02] (ns)	0.01 [−0.0, 0.03] (ns)	−0.0 [−0.02, 0.01] (ns)
	***central***	−0.0 [−0.01, 0.01] (ns)	0.01 [−0.0, 0.02] (ns)	−0.01 [−0.02, 0.0] (ns)
	***parietal***	**−0.01 [−0.01**, −0.0] (*)	0.0 [−0.0, 0.01] (ns)	**−0.01 [−0.02**, −0.0] (*)
	***occipital***	**−0.02 [−0.02**, −0.01] (*)	0.0 [−0.01, 0.01] (ns)	**−0.02 [−0.03**, −0.01] (*)
***alpha peak***	***overall***	**−0.5 [−0.85**, −0.15] (*)	0.4 [−0.01, 0.81] (ns)	**−0.9 [−1.42**, −0.37] (**)
	***frontal***	**−0.31 [−0.79**, −0.17] (**)	0.32 [−0.13, 0.73] (ns)	**−0.8 [−1.25**, −0.29] (**)
	***central***	**−0.28 [−0.89**, −0.25] (***)	**0.43 [0.05**, 0.85] (*)	**−1 [−1.48**, −0.54] (***)
	***parietal***	**−0.28 [−0.89**, −0.25] (***)	**0.44 [0.02**, 0.84] (*)	**−1 [−1.47**, −0.55] (***)
	***occipital***	**−0.29 [−0.75**, −0.09] (*)	**0.45 [0.05**, 0.86] (*)	**−0.88 [−1.35**, −0.41] (***)
***STFR***	***overall***	−0.0 [−0.23, 0.23] (ns)	−0.18 [−0.42, 0.07] (ns)	0.18 [−0.15, 0.48] (ns)
	***frontal***	−0.0 [−0.34, 0.33] (ns)	−0.26 [−0.6, 0.13] (ns)	0.25 [−0.19, 0.65] (ns)
	***central***	0.02 [−0.22, 0.28] (ns)	−0.23 [−0.48, 0.05] (ns)	0.25 [−0.05, 0.57] (ns)
	***parietal***	0.03 [−0.17, 0.26] (ns)	−0.15 [−0.36, 0.1] (ns)	0.17 [−0.11, 0.45] (ns)
	***occipital***	−0.03 [−0.23, 0.14] (ns)	−0.17 [−0.36, 0.02] (ns)	0.13 [−0.09, 0.35] (ns)

For each group comparison the Median difference (MD) and the corresponding Highest-density interval (HDI) of the fitted coefficients are shown. Statistically significant values (HDI not containing 0, *p* < 0.05) are marked with an asterisk depending on the degree of significance (*: *p* < 0.05, **: *p* < 0.01, ***: *P* < 0.001) and highlighted in bold. HC: Healthy controls; ns: not significant; STFR: slow-to-fast ratio

Differences in relative power among the groups, are shown in Fig. 2; Table 2. Looking at regional differences, the most striking differences concerned again the theta band. Indeed, patients with *CACNA1A* disease displayed significantly higher median power values in the theta band than in healthy controls and GAA-*FGF14* patients in all studied brain regions (Fig. 2b). Additionally, *CACNA1A* patients displayed decreased median power values in the gamma band in the parietal and occipital regions as compared to both the control and GAA-*FGF14* groups (Fig. 2e).

Findings in GAA-*FGF14* patients largely overlapped with those of the control group. Notably, GAA-*FGF14* patients showed a significant increase in median beta power compared to healthy controls in both the parietal and occipital regions (Fig. 2d). No significant differences were found in the delta and alpha bands (Fig. 2a and c) and in the slow-to-fast ratio (Supplemental Fig. 1) across the three groups.

### Connectivity

**Fig. 3:**
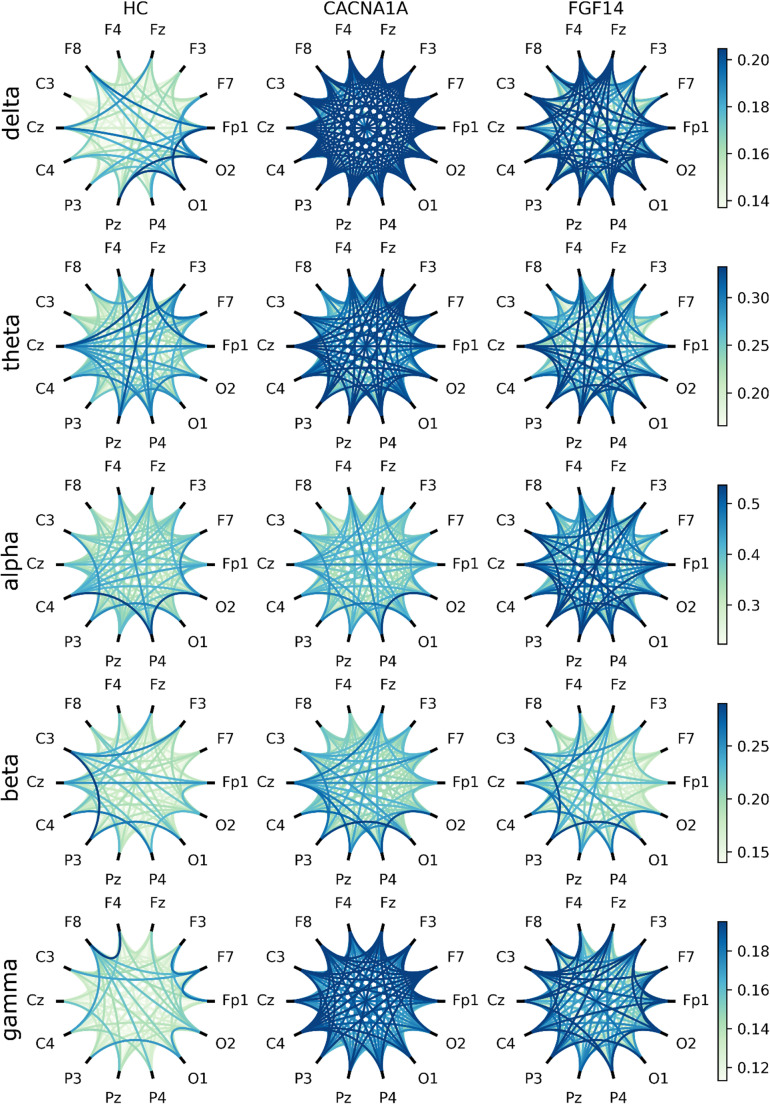
Graphical representation of the connectivity between electrodes: Average weighted phase-lag index (wPLI) values calculated for each frequency band and between each electrode pair. HC: healthy controls

**Fig. 4:**
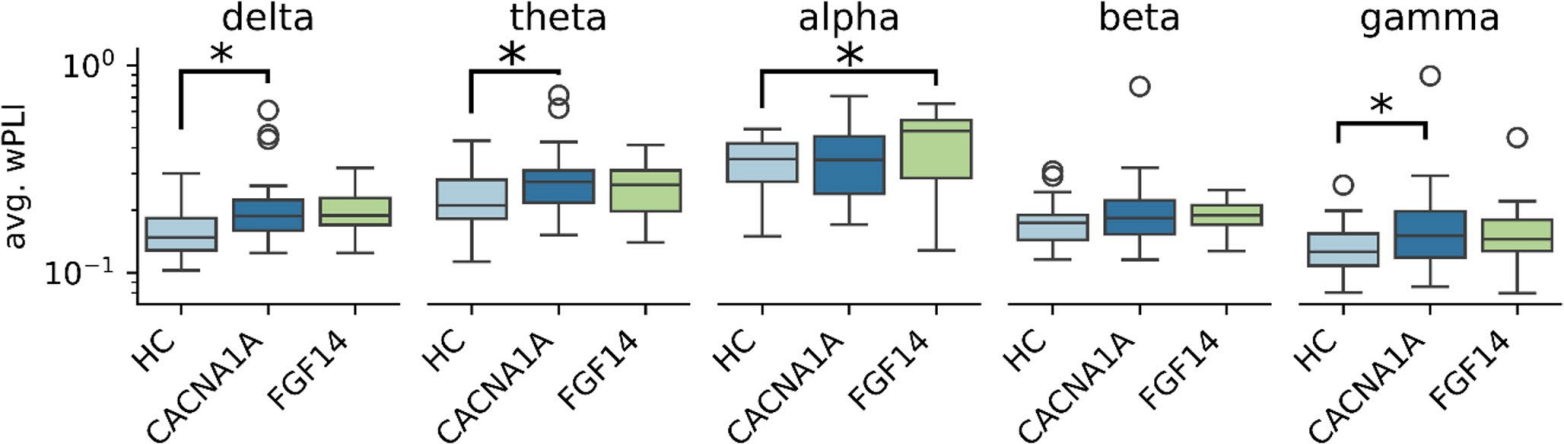
Results of connectivity analysis: weighted phase-lag index averaged over all connections (avg. wPLI). Values are shown as logit-transformed. *: *p* < 0.05

A graphical overview of average functional connectivity between electrodes, estimated using the wPLI metric, is presented in Fig. 3, where connection strength between electrodes is visualized. Group differences derived from the wPLI-weighted connectivity graph are summarized in Table 3 using MST metrics. Several differences in average wPLI were observed across both low- and high-frequency bands (Fig. 4). *CACNA1A* patients exhibited increased average connectivity in the delta/theta and gamma bands compared with healthy controls. In contrast, GAA-*FGF14* patients showed increased average connectivity in the alpha band relative to controls. No significant differences were observed for the MST metrics meaning Diameter, Maximum Betweenness Centrality and Leaf Fraction across the three groups (Supplemental Fig. 2).

**Table 3:** Connectivity analysis findings

		CACNA1A vs. HC	FGF14 vs. HC	CACNA1A vs. FGF14
variable	band	MD [HDI]	MD [HDI]	MD [HDI]
***wPLI***	***delta***	**0.04 [0.02**, 0.07] (*)	0.03 [−0.0, 0.06] (ns)	0.01 [−0.03, 0.05] (ns)
	***theta***	**0.05 [0.02**, 0.1] (*)	0.03 [−0.01, 0.07] (ns)	0.02 [−0.03, 0.08] (ns)
	***alpha***	0.04 [−0.02, 0.08] (ns)	**0.06 [0.01**, 0.12] (*)	−0.02 [−0.09, 0.04] (ns)
	***beta***	0.03 [−0.0, 0.06] (ns)	0.02 [−0.02, 0.06] (ns)	0.0 [−0.04, 0.05] (ns)
	***gamma***	**0.03 [0.01**, 0.06] (*)	0.03 [−0.0, 0.06] (ns)	0.01 [−0.03, 0.04] (ns)
***Diameter***	***delta***	−0.01 [−0.04, 0.01] (ns)	−0.03 [−0.06, 0.0] (ns)	0.01 [−0.02, 0.05] (ns)
	***theta***	−0.01 [−0.03, 0.01] (ns)	−0.02 [−0.05, 0.01] (ns)	0.01 [−0.02, 0.05] (ns)
	***alpha***	−0.01 [−0.03, 0.01] (ns)	−0.02 [−0.05, 0.01] (ns)	0.01 [−0.02, 0.05] (ns)
	***beta***	−0.01 [−0.03, 0.02] (ns)	−0.02 [−0.06, 0.01] (ns)	0.02 [−0.02, 0.05] (ns)
	***gamma***	−0.01 [−0.03, 0.01] (ns)	−0.02 [−0.05, 0.02] (ns)	0.01 [−0.04, 0.04] (ns)
***MBC***	***delta***	0.02 [−0.01, 0.06] (ns)	0.01 [−0.03, 0.05] (ns)	0.01 [−0.04, 0.06] (ns)
	***theta***	0.01 [−0.02, 0.04] (ns)	0.0 [−0.04, 0.04] (ns)	0.01 [−0.04, 0.05] (ns)
	***alpha***	0.01 [−0.02, 0.03] (ns)	0.01 [−0.03, 0.05] (ns)	−0.0 [−0.05, 0.04] (ns)
	***beta***	0.01 [−0.02, 0.03] (ns)	0.01 [−0.02, 0.06] (ns)	−0.01 [−0.06, 0.04] (ns)
	***gamma***	0.01 [−0.02, 0.03] (ns)	−0.01 [−0.06, 0.03] (ns)	0.02 [−0.03, 0.07] (ns)
***Leaf*** ***Fraction***	***delta***	0.02 [−0.01, 0.06] (ns)	0.02 [−0.02, 0.07] (ns)	0.0 [−0.05, 0.06] (ns)
	***theta***	0.01 [−0.02, 0.04] (ns)	0.01 [−0.03, 0.06] (ns)	−0.01 [−0.06, 0.05] (ns)
	***alpha***	0.0 [−0.03, 0.03] (ns)	0.02 [−0.02, 0.06] (ns)	−0.02 [−0.07, 0.03] (ns)
	***beta***	0.0 [−0.03, 0.03] (ns)	0.03 [−0.02, 0.08] (ns)	−0.03 [−0.09, 0.02] (ns)
	***gamma***	0.01 [−0.03, 0.04] (ns)	0.0 [−0.05, 0.05] (ns)	0.0 [−0.05, 0.07] (ns)

For each group comparison the Median difference (MD) and the corresponding Highest-density interval (HDI) of the fitted coefficients are shown. Values are rounded to 2 digits. Statistically significant values (HDI not containing 0, *p* < 0.05) are marked with an asterisk and highlighted in bold. Abbreviations: HC: Healthy controls; ns: not significant; wPLI: weighted Phase-lag Index; MBC: Maximum betweenness centrality.

## Discussion

This monocentric study provides insights on the electrophysiological signature associated with two rare neurogenetic channelopathies: *CACNA1A*- and GAA-*FGF14*-related disorders. Using quantitative rsEEG analysis methods and Bayesian hierarchical modeling, we identified meaningful differences in spectral power and connectivity patterns among *CACNA1A* patients, GAA-*FGF14* patients and healthy controls. Considering spectral power analysis, *CACNA1A* patients displayed an increased power in theta band and a lower alpha peak frequency as compared with healthy controls across all the brain regions. In contrast, findings from bandpower analysis in GAA-*FGF14* patients largely overlapped with those of healthy controls. Considering connectivity, *CACNA1A* patients again displayed several differences compared with healthy controls consisting of increased connectivity parameters in both lower and higher frequency bands. GAA-*FGF14* patients displayed a hyperconnectivity in the alpha band as compared with controls.

*CACNA1A*-associated disease was the most frequent etiology in a large global cohort of patient with cerebellar ataxias due to non-expansion variants [2] and is increasingly recognized in the setting of manifold neurological phenotypes [10]. Recently, international collaborations between clinicians and patient representatives have been established, fostering progress toward addressing critical unresolved issues in the field [29]. First, the pathophysiological mechanisms underlying the extreme clinical variability, even among individuals within the same family, remain largely unknown. In turn, this variability poses a significant challenge in establishing appropriate and sensitive endpoints to assess disease burden in natural history studies and clinical trials. Conventional clinician-reported outcome measures may fail to adequately capture the morbidity associated with the manifold clinical presentations and progression patterns [2]. This particularly apply when considering the neuropsychiatric and cognitive symptoms, which are heterogeneous and not captured in their entirety by the currently available scales [30]. Although variants associated with more severe phenotypes are known to cause more pronounced alterations in Ca^2+^ current dynamics, no surrogate biomarkers currently exist that reliably reflect these functional abnormalities. RsEEG is a quick, non-invasive, inexpensive investigation, which is usually available in every neurology clinic. Advanced rsEEG analysis has been used as a non-invasive methodology to assess cortical dynamics and network dysfunction in a variety of neurological disorders [13, 15, 16]. More importantly, rsEEG is a functional assessment reflecting dynamic neuronal processes in the time frame in which these occur. Thus, rsEEG signal alterations upon various conditions, including therapeutic interventions, have the potential to reflect a patient-relevant change in disease state. In the present study, quantitative rsEEG analysis methods detected in *CACNA1A* patients changes (e.g. increased power in lower frequency bands) which appear to be consistent with the alterations observable at naked eye (frequent lateralized intermittent slowing, see also [9]). These findings in turn may represent a proxy of the brain dysfunction underlying cognitive and neuropsychiatric symptoms in *CACNA1A* patients. Differently from the routine examination at naked eye, advanced rsEEG analysis offers an objective, quantitative assessment that allows comparison and reproducibility in larger, multicentric cohorts and is thus potentially suitable as surrogate marker.

The most evident alterations in rsEEG signals in *CACNA1A* patients were a reduced alpha peak frequency and an increase in theta bandpower. Alpha peak frequency is an electrophysiological trait that has been linked to cognitive performance; lower frequencies are associated with impaired cognition and ageing [31]. Theta oscillation is one of the largest and most sinusoidal activity patterns in the brain. Experimental evidence links theta rhythm to information processing, memory and learning [32]. Increased theta oscillations have been observed as non-specific finding in a variety of neurological and psychiatric disorders [33–35] and may reflect a state of “thalamocortical dysrhythmia” [33]. This early postulated model was based on the observation that disruption of thalamic inputs in various pathological conditions leads to a switch in the firing pattern of thalamocortical neurons and subsequently entrains thalamic and cortical areas with pathological oscillations in the theta range [33]. Notably, P/Q Ca2 + channel knockout mice were established early on as a model of thalamocortical dysrhythmia [36]. EEGs were recorded during wakefulness and visually inspected for vigilance-related changes. Those performed during or up to one week after spell episodes were excluded. Therefore, we are confident that the increase in theta activity in *CACNA1A* patients does not reflect fluctuations in vigilance.

Furthermore, *CACNA1A* patients displayed a hyperconnectivity pattern across both low and high frequency bands. The pathophysiological interpretation of these findings alone is challenging. In multimodal studies from “network disorders” such as Alzheimer´s disease and schizophrenia both increasing and decreasing synchronization correlate with cognitive deterioration [14, 37–39]. Connectivity was also evaluated via MST metrics, recently proposed graph-theory based parameters which have been developed to overcome the drawbacks of comparisons between raw connectivity values such as poor reproducibility [40, 41]. These metrics reflect only the strongest connections between nodes and not the average connectivity strength, thus increasing robustness. In our dataset however, groups did not differ in their connectivity structure but rather in the overall connectivity of the whole network, a phenomenon not picked up by MST metrics. Furthermore, it could be argued that the electrode number used in this study was too low to find meaningful differences in network topology. Future studies using higher density EEG recordings could clarify this issue.

In the present study, we compared rsEEG of *CACNA1A* patients also with those of patients with GAA-*FGF14* disease. This study design was motivated by the overlapping clinical presentation of specific *CACNA1A* disease phenotypes with GAA-*FGF14* ataxia [42]. In a previous work of our group on visual analysis of routine EEGs, we compared *CACNA1A* patients with clinical “phenocopies” [9]. Several of them were recently diagnosed with GAA-*FGF14* disease. This led us to investigate whether rsEEG analysis may discriminate disease-specific electrophysiological footprints of the two disorders. In fact, *CACNA1A* patients displayed numerous differences both in the spectral and connectivity analysis compared with GAA-*FGF14* patients, who, on the other hand, did show largely overlapping findings with healthy controls in bandpower analysis. Overall, these findings seem consistent with the clinical observation of a rather pure motor disorder in GAA-*FGF14*-related disease, as opposed to *CACNA1A* variants, which often result in prominent, additional non-motor symptoms, as likely result of more extensive network alterations. Quantifying and translating this dysfunction into a biomarker is an open, challenging issue in *CACNA1A* research [29]. Since structural imaging does not show obvious neuroanatomical changes outside the cerebellum in *CACNA1A* disorders, functional investigations appear the most promising approach in the quest for surrogate biomarkers.

The present study has a number of limitations. Control subjects EEG were retrieved from an open access database (not in-house controls) and thus recorded with different hardwares, although they underwent the same pre-processing of the EEG recorded from patients with *CACNA1A*- and GAA-*FGF14-*related disease. Furthermore, there were significant differences in terms of age among the considered groups, which we addressed by fitting a hierarchical Bayesian model that incorporates priors for the age coefficients estimated from an independent population of healthy controls. This approach allowed us to model the expected influence of age on the outcome variable separately from the group effect. Additionally, the incorporation of prior knowledge and accounting for the relationship between power and connectivity metrics increases detection power and eliminates the need for correction for multiple comparisons [23]. This study had a pilot design with a limited patient count from a single center, thus preventing us from investigating genotype-phenotype correlations, e.g. across different *CACNA1A* variants, or correlations between rsEEG pattern and other disease milestones (e.g. cognitive performance, severity of motor syndrome) and medication use. Furthermore, future prospective studies with standardized experimental conditions may explore further aspects of EEG in these channelopathies, e.g. vigilance-related changes in comparison to controls. Finally, the underrepresentation of females in our study is a notable limitation. This merely mirrors the sex distribution of the cohort followed at our institution and constrains the generalizability of our findings to *CACNA1A*- and GAA-*FGF14*-related channelopathies in their entirety.

## Conclusions

We employed advanced rsEEG analysis to study the electrophysiological signature of *CACNA1A-* and GAA-*FGF14-*related disorders, two rare neurogenetic channelopathies. This approach enabled us to identify disease-specific electrophysiological signatures that distinguish these two conditions from each other and from healthy controls. Encouraged by these preliminary findings, we plan to apply the developed algorithm to a larger, multicenter *CACNA1A* cohort to refine our results and further explore its potential for clinical application in natural history studies or interventional trials.

## Supplementary Information

Below is the link to the electronic supplementary material.

Supplementary Material 1 (DOCX 194 KB)

## Data Availability

The dataset supporting the present findings is available from the corresponding author upon reasonable request. The code used for rsEEG analysis is publicly available.
