# Supplementary material for: Resting-State EEG Analysis Characterizes the Signature of CACNA1A-and GAA-FGF14-Related Channelopathies
Source: Cerebellum. 2025 Nov 15;24(6):175. doi: 10.1007/s12311-025-01924-w (PMC12619801; doi:10.1007/s12311-025-01924-w)
Supplement: Supplementary file 1 — Supplementary Material 1 (DOCX 194 KB) [file 12311_2025_1924_MOESM1_ESM.docx]

**Supplemental Methods**

**Construction of the Bandpower Model**

A naive approach would be to model each EEG frequency band separately. However, this ignores the fact that the relative bandpowers across all bands in a given EEG segment always sum to one. This constraint means the bands are not independent but are negatively correlated. Mathematically, the vector of relative bandpowers

$$Y:=(y_{\delta},\ldots,y_{\gamma})$$

does not span the whole five-dimensional real space , but instead lies in a *simplex* (a constrained space where components sum to one) [Greenacre, 2021].

To analyze RBPs correctly, we need to “open up” this simplex into an unconstrained space. This can be achieved by transforming the data into log-ratios $L(Y)$. At each brain region l, these four log-ratios were modeled as coming from a multivariate normal distribution with mean vector

$$\hat{\mu}_{l}:=(\mu_{l,1},\ldots,\mu_{l,4})$$

and covariance matrix $\Sigma$:

$$L\left( Y_{l} \right)\mathcal{\sim N(}\hat{\mu}_{l},\Sigma)$$

The covariance structure was split into two components: a diagonal matrix of standard deviations $S$ with entries drawn from an exponential prior, ${(S)}_{i,i} \sim Exp(1)$, and a correlation matrix $C$. The prior for $C$ followed the Lewandowski–Kurowicka–Joe (LJK) distribution:

$$\Sigma=SCS$$

The mean of each log-ratio, $\mu_{l,r}$​, was further explained using a linear regression model.

$$\mu_{l,r}=\hat{\beta}_{l,r}X_{l,r}$$

$$\hat{\beta}_{l,r}=(\beta_{l,r}^{Intercept},\beta_{l,r}^{Age},\beta_{l,r}^{Sex},\beta_{l,r}^{CACNA1A},\beta_{l,r}^{FGF14})$$

To capture correlations across brain regions, regression coefficients were modeled hierarchically. Specifically, each region-specific coefficient $\beta_{l,r}$ was assumed to vary around a shared global effect $\tilde{\beta}_{r}$​:

$$\beta_{l,r}=\tilde{\beta}_{r}+\sigma_{r}z_{l,r}$$

$$z_{l,r}\mathcal{\sim N}(0,1)$$

Here, $\hat{\beta}_{r}$​ reflects the overall effect of a predictor across all regions, while the term $\sigma_{r}z_{l,r}$ allows for region-specific deviations.

**Construction of the connectivity model**

We applied a similar strategy when modeling connectivity measures. For each frequency band $b$, we considered the vector

$$Y:=(y_{wPLI},y_{Dia},y_{LF},y_{MBC})$$

which includes the weighted Phase Lag Index (wPLI) and three Minimum Spanning Tree (MST)-based measures: diameter (Dia), leaf fraction (LF), and mean betweenness centrality (MBC). This vector was modeled as multivariate normal:

$$Y_{b}\mathcal{\sim N(}\hat{\mu}_{b},\Sigma)$$

As in the standard approach, wPLI values were logit-transformed. Correlations among the MST metrics were expected to be strong, since these measures are mathematically interdependent. For example, a graph with maximum diameter (where nodes are arranged in a line) inevitably has low leaf fraction and reduced mean betweenness centrality.

To quantify these correlations, we generated 10,000 synthetic fully connected graphs with the same number of nodes as electrodes. Edge weights were drawn randomly from a uniform distribution $w\sim\mathcal{U}(0,1)$, which mimicked wPLI values. We then computed the MST for each graph and derived the corresponding metrics. This procedure allowed us to empirically estimate the covariance structure $\Sigma$, which was decomposed as described above and used the same priors for standard deviations.

The mean vector for each frequency band was defined as

$$\hat{\mu}_{b}:=(\mu_{b,1},\ldots,\mu_{b,4})$$

with each element modeled using linear regression.

$$\mu_{b,m}=\hat{\beta}_{b,m}X_{b,m}$$

$$\hat{\beta}_{b,m}=(\beta_{b,m}^{Intercept},\beta_{b,m}^{Age},\beta_{b,m}^{Sex},\beta_{b,m}^{CACNA1A},\beta_{b,m}^{FGF14})$$

To stabilize estimation and account for similarities between metrics, regression coefficients were again given a hierarchical structure:

$$\beta_{b,m}=\tilde{\beta}_{m}+\sigma_{b}z_{b,m}$$

$$z_{b.m}\mathcal{\sim N}(0,1)$$

In this case, however, the shared mean $\tilde{\beta}_{m}$ does not have a direct interpretation (unlike in the relative bandpower model). It was included purely to provide regularization, preventing coefficients from drifting too far apart across bands.

**Supplemental Figures**


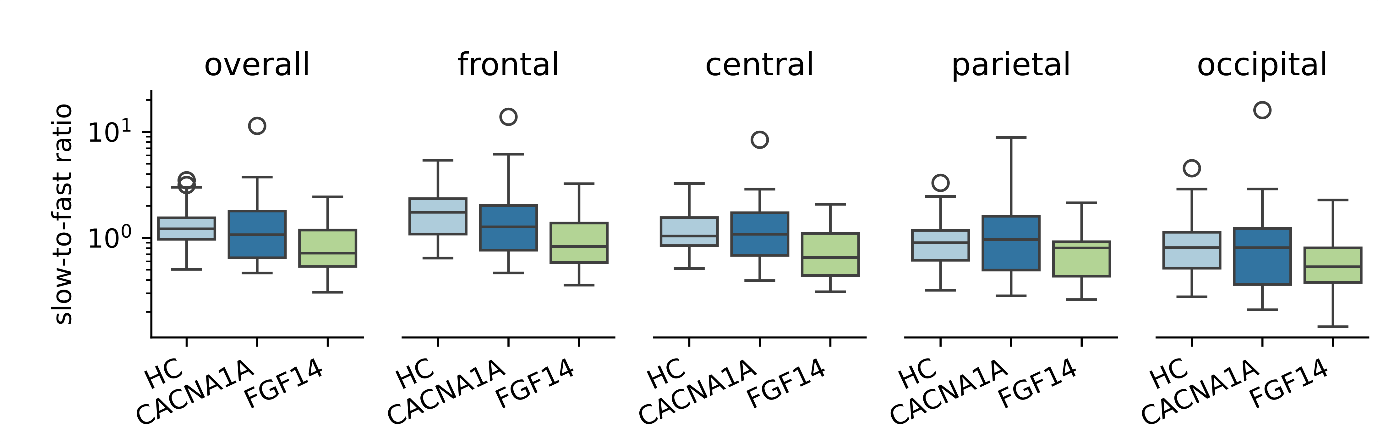


**Supplemental Figure 1:** **Results of slow-to-fast ratio analysis**: Log-transformed slow-to-fast ratio values. Abbreviations: HC: Healthy controls, *: p < 0.05, **: p < 0.01, ***: p<0.001


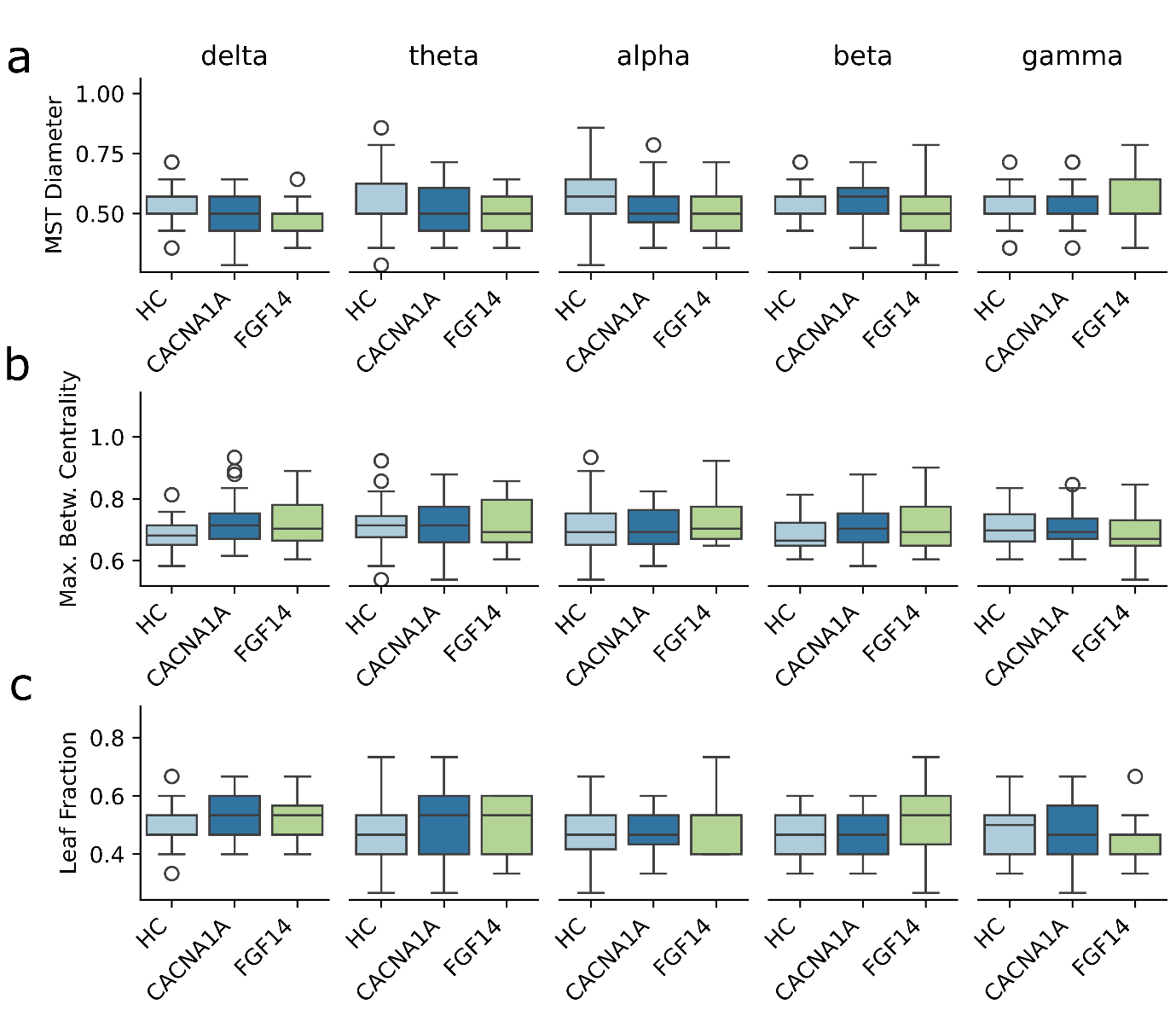


**Supplemental Figure 2:** **Results of MST-metrics analysis**: Minimum-spanning tree (MST) metrics: Diameter (a), Maximum betweenness centrality (b) and Leaf Fraction (c). Abbreviations: HC: Healthy controls, *: p < 0.05, **: p < 0.01, ***: p<0.001
